# Effects of Curing Conditions on Splitting Tensile Behavior and Microstructure of Cemented Aeolian Sand Reinforced with Polypropylene Fiber

**DOI:** 10.3390/ma16196347

**Published:** 2023-09-22

**Authors:** Bo Ruan, Tianyao Zhou, Zhongzheng Yuan, Jenisha Singh, Jidong Teng, Shilong Zheng, Jiasen Zhang

**Affiliations:** 1 School of Civil Engineering, Central South University, Changsha 410075, China; ruanbo@csu.edu.cn (B.R.); 224812370@csu.edu.cn (T.Z.); 214811125@csu.edu.cn (Z.Y.); jenishasingh2022@gmail.com (J.S.); 214811113@csu.edu.cn (J.Z.); 2 National Engineering Research Centre for High-Speed-Railway Construction, Central South University, Changsha 410075, China; 3 China Railway SiYuan Survey and Design Group Co., Ltd., Wuhan 430063, China; 007448@crfsdi.com

**Keywords:** cemented aeolian sand, polypropylene fiber, curing temperature, fiber content, STS, NMR

## Abstract

Aeolian sand is widely distributed in the Takramagan Desert, Xinjiang, China, which cannot be directly used as railway subgrade filling. It is beneficial for environmental protection to use fiber and cement-reinforced aeolian sand as railway subgrade filling. The present work is to explore the enhancement of tensile strength in cemented aeolian sand via the incorporation of polypropylene fibers under conditions of elevated temperature and drying curing. The purpose Is to delve into the examination of the temperature’s impact on not only the mechanical attributes but also the microstructure of cemented aeolian sand reinforced with polypropylene fiber (CSRPF). For this, a comprehensive set of tests encompassing splitting tensile strength (STS) assessments and nuclear magnetic resonance (NMR) examinations is conducted. A total of 252 CSRPF specimens with varying fiber content (0, 6‰, 8‰, and 10‰) are tested at different curing temperatures (30 °C, 40 °C, 50 °C, 60 °C, 70 °C, and 80 °C). The outcomes of the NMR examinations indicate that elevating the curing temperature induces the expansion of pores within CSRPF, both in size and volume, consequently contributing to heightened internal structural deterioration. STS tests demonstrate that the STS of CSRPF decreases as the curing temperature increases. Meanwhile, the STS of CSRPF increases with fiber content, with optimal fiber content being 8‰. Regression models accurately predict the STS, with the curing temperature exhibiting the greatest influence, followed by the fiber content according to sensitivity analysis. The research results provide a valuable reference for the use of CSRPF as railway subgrade filling under high temperature and drying conditions.

## 1. Introduction

The Hotan-Ruoqiang Railway (abbreviated as Heruo Railway) extends from west to east along the southern edge of the Taklimakan Desert in Xinjiang. Aeolian sand is widely distributed on the surface of the Taklimakan Desert through which the Heruo Railway passes. The surface of aeolian sand is smooth, non-sticky, can be easily blown by the wind, and has uniform particle size and poor gradation [1,2,3]. According to China’s national criterion for high-speed railway design, the aeolian sand is classified as gap-graded C3 filler. However, its direct use as subgrade filling for high-speed railways is not recommended. Before considering the aeolian sand as a viable option for high-speed railway subgrades, it is essential to enhance its physical and chemical properties. This proactive approach will ensure that the aeolian sand meets the necessary criteria for optimal performance within the context of high-speed railway infrastructure. Since there is a lack of high-quality fill materials along the Heruo Railway, the abundant and inexpensive local aeolian sand is used as a substitute for the conventional fill materials. This reduces environmental damage, lowers construction costs, shortens construction time, and provides better economic and ecological benefits.

Cement serves as a valuable admixture in engineering contexts, playing a pivotal role in enhancing the physical and mechanical characteristics of soils. When soil is cemented, it exhibits remarkable strength and stiffness, rendering it a favored choice for diverse applications such as railway embankments, highway constructions, and numerous other engineering applications [4,5]. However, cemented soil is more brittle and has lower crack resistance. After the fracture, the soil quickly loses its load-bearing capacity, which can easily lead to engineering accidents [6,7,8]. In addition, the large-scale use of cement promotes the emission of carbon dioxide and causes environmental pollution [9], which is contrary to the measures advocated by China and the whole world to reduce carbon dioxide emissions and protect the environment. Therefore, enhancing the crack resistance of soil and reducing the use of cement are the main problems of current soil improvement technology. In recent times, a multitude of studies have advocated for the incorporation of both fibers and cement into soil matrices. This innovative approach aims to elevate the overall physical and mechanical attributes of the soil, leading to a substantial enhancement in its performance and suitability for various applications [10,11,12,13]. Cemented soil reinforced with fiber is an important development direction of soil improvement technology. The addition of fibers in cemented soils not only helps in the enhancement of mechanical properties (strength and crack resistance) [9,14,15,16] but also reduces the amount of cement used, leading to a reduction in carbon emissions and environmental pollution.

Many factors influence the reinforcement effect of fiber in the soil [17,18,19]. The reinforcement impact of fibers is directly influenced by two important parameters: fiber content and fiber length. Introducing a small amount of fibers or utilizing short fibers in the soil yields negligible effects on soil reinforcement. Conversely, an excess of fibers or fibers that are excessively long impedes the reinforcement potential within the soil. Thus, an optimal equilibrium is reached by determining the appropriate fiber content and fiber length. The optimal value corresponds to a scenario where the fiber content and fiber length synergistically enable the complete manifestation of the reinforcing influence of the fibers [20,21,22,23]. A multitude of experimental investigations has demonstrated that the ideal fiber content hinges on both the fiber variety and the soil type, as indicated by numerous empirical studies [24,25,26]. When polypropylene fiber is used to reinforce clay and tailings, the optimum fiber content is 3‰ and 1.5‰ [20,24]. When glass fiber is used to reinforce clay and tailings, the optimum fiber content is 10‰ and 6‰ [27,28]. Nonetheless, the optimal fiber length remains consistent irrespective of the fiber and soil type. Typically, the optimal fiber length is around 6 mm [20,22,29].

Polypropylene fiber is a synthetic chemical fiber made from isotactic polypropylene. Polypropylene fiber finds extensive application in engineering endeavors due to its notable attributes, including elevated tensile strength, commendable resilience, lightweight composition, robust resistance to corrosion, economical cost, and environmentally conscious characteristics [30,31,32]. The inclusion of polypropylene fiber does not exert a noteworthy influence on the maximum dry density and the optimal moisture content [6], but it enhances both the fiber content and length, yielding more substantial benefits in bolstering the strength and ductility of polypropylene fiber-reinforced cemented soil. The impact of elevated fiber content on soil reinforcement outweighs that of varying fiber length, yet it remains less potent than the influence of increasing cement content [20,30]. The single polypropylene fiber has better performance than the polypropylene fiber bundle [31]. Polypropylene fiber can effectively minimize the generation of cracks. The specimens with higher fiber content have higher crack resistance and fewer cracks [24]. Compared with other fibers, polypropylene fiber has the most significant effect on soil reinforcement at the same fiber content [26]. Therefore, polypropylene fiber is used to reinforce aeolian sand in the Taklimakan Desert in Xinjiang, and the research results have important engineering value for the application of aeolian sand.

The Taklimakan Desert, located in the northwestern part of China, is in a dry climate zone. The annual mean temperature difference generally exceeds 30 °C, and the absolute temperature difference can even reach more than 50 °C. Summer is extremely hot and the near-surface temperature can reach 70 °C at noon in summer. There is very little rainfall throughout the year, with an average annual rainfall of less than 50 mm. The desert area has high evaporation, and the average annual evaporation of the Taklimakan Desert reaches 3000 mm. The air in the Taklimakan Desert is dry and humidity can be as low as 5%. The hydration kinetics of cement is closely related to the curing temperature, and different curing temperatures have a significant impact on the mechanical properties of cement–matrix composites [33,34,35]. Raising the curing temperature results in a reduction of both the strength and ductility of the cement–matrix composite while simultaneously increasing its elastic modulus. This effect is more prominent with higher cement content, as indicated by previous studies [36]. Nevertheless, conflicting perspectives suggest that the curing temperature exhibits an opposite influence during the initial curing stages [36,37,38,39]. In essence, the curing temperature serves as a pivotal element influencing the mechanical properties of cemented aeolian sand reinforced with polypropylene fibers (CSRPF). Considering the climatic characteristics of the Taklimakan Desert, when railway construction is carried out in summer, the maintenance of CSRPF is susceptible to environmental temperature.

An array of nuclear magnetic resonance (NMR) and (STS) examinations were conducted to scrutinize the impact of curing temperature on the internal distribution of pore sizes and the mechanical attributes of CSRPF. The outcomes of this research furnish valuable perspectives for the planning and execution of railway line projects that incorporate CSRPF as a subgrade bed filling material.

## 2. Materials and Methods

### 2.1. Materials

**Figure 1:**
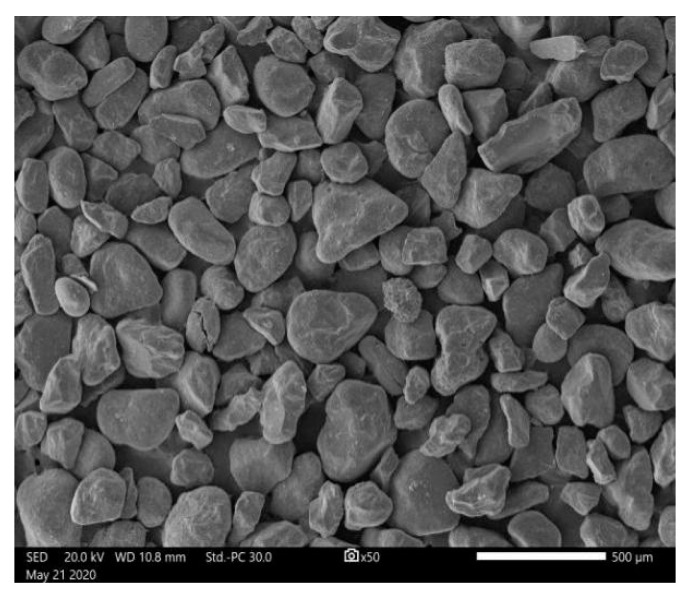
Scanning electron micrograph of aeolian sand.

**Figure 2:**
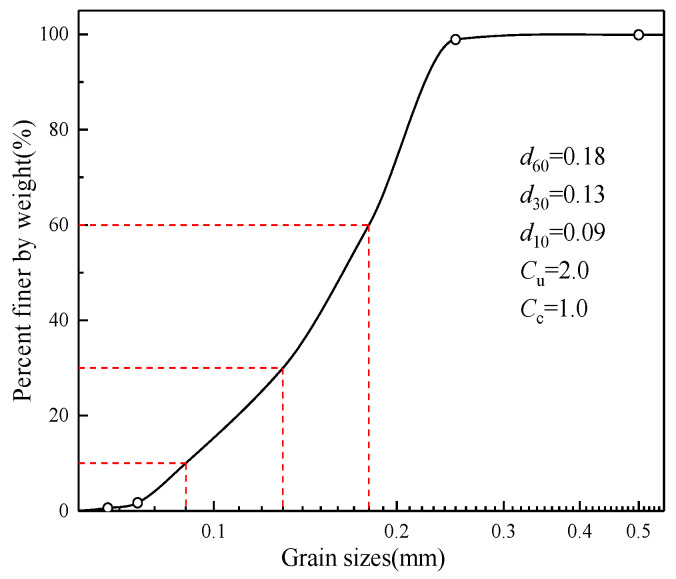
Grain size distribution of the aeolian sand.

**Figure 3:**
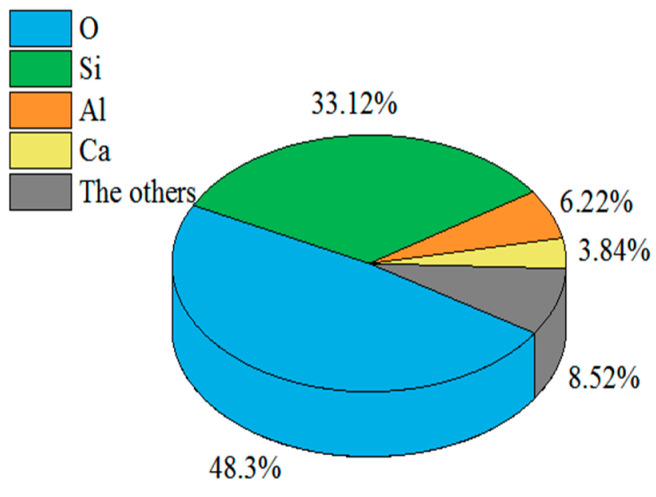
Chemical element ratio of the aeolian sand.

Aeolian sand was sampled from a railway construction site of the Heruo Railway in the Taklimakan Desert in Xinjiang, China. Figure 1 shows the 50× magnified scanning electron micrograph of the tested aeolian sand. Figure 2 illustrates the grain size distribution curve of the aeolian sand. The predominant grain size range falls within 0.075 to 0.25 mm, constituting 97.2% of the total grain distribution. The particles of aeolian sand are evenly distributed and have poor gradation. Figure 3 shows the chemical composition of aeolian sand. The two elements O and Si in aeolian sand account for the largest proportion, indicating that its main component is SiO_2_, and other components are Al_2_O_3_, CaO, and MgO. The properties of aeolian sand are summarized in Table 1.

Ordinary Portland cement was utilized as the primary binding element; its chemical composition is shown in Table 2. The resultant cement mortar displayed an average compressive strength of 18 MPa at the end of 3 days, which increased to 45 MPa after a 28-day period.

**Figure 4:**
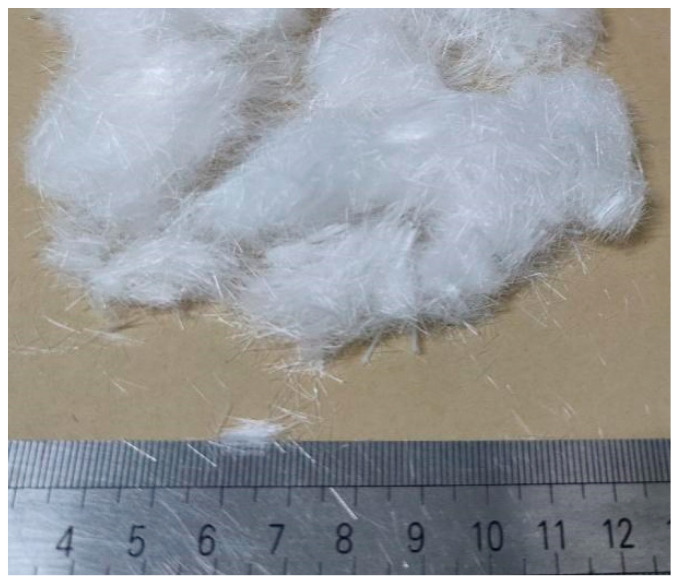
Photograph showing the discrete polypropylene fibers.

Polypropylene fiber (Figure 4) was chosen as the reinforcing material due to its remarkable dispersibility, elevated tensile strength, superior elastic modulus, and environmentally conscious attributes [40]. Certain attributes of the polypropylene fibers, as supplied by the manufacturer, have been detailed in Table 3.

### 2.2. Test Scheme

The STS specimens and NMR specimens were 28 groups each. Each STS test group contained 6 specimens, with a subtotal of 168 specimens. There were 3 samples in each group of the NMR test, with a subtotal of 84 samples. There were 252 samples in total.

The cement content, *a_c_*, and the fiber content, *a_f_*, are defined as follows in Equation (1):

(1) $$a_{c}=\frac{m_{c}}{m_{s}}\times 100\%\;a_{f}=\frac{m_{f}}{m_{s}}\times 100\%$$

where *m_c_* is the quality of dry cement, *m_s_* is the quality of dry aeolian sand, and *m_f_* is the quality of fiber.

The control group consisted of specimens subjected to standard curing conditions. The remaining specimens underwent curing at distinct temperatures, ranging from 30 °C to 80 °C, with an incremental increase of 10 °C. Four distinct fiber contents were configured at 0‰, 6‰, 8‰, and 10‰, while the cement content was maintained at 5%. The curing period was established at 7 days, accompanied by a compaction coefficient of 0.95, representing the ratio of the dry density of specimens to the maximum dry density.

Since the surface temperature in the Taklimakan desert can reach 70 °C at noon in summer, the curing temperatures in the test scheme were set with an interval of 10 °C. The initial testing revealed that fibers tend to aggregate, forming larger clusters during specimen preparation when the fiber content exceeds 10‰. As a result, the upper limit for the fiber content was established at 10‰. The cement content, curing age, and compaction coefficient were determined according to the Code for Design of Railway Earth Structure (TB 10001-2016) [41] (i.e., a national criterion for high-speed railway design in China). The fiber lengths are consistent with previous studies [20,42], allowing comparisons with previous results.

### 2.3. Preparation of the Specimens

The oven-dried aeolian sand was weighed along with water to determine the values for maximum dry density and optimum moisture content. First, the polypropylene fiber was slowly mixed into the aeolian sand. The fibers and the aeolian sand were mixed and scattered by hand, and the scattered fibers and the aeolian sand were evenly mixed and stirred to prevent the fibers from agglomerating. Then, cement was added to the mixture of fiber and aeolian sand and mixed evenly. Subsequently, water was introduced into the blend and meticulously combined using an electric mixer.

The compound was carefully poured into molds possessing a diameter of 50 mm and a height of 100 mm. Employing the static compaction method outlined in the Code for Soil Test of Railway Engineering [43] (TB 10102-2010), the specimen was compacted. Each specimen, with a diameter and height of 50 mm, was carefully extracted from the mold and subsequently enveloped with a protective plastic membrane. Subsequently, two distinct sets of specimens were subjected to curing protocols. The initial group underwent a curing process within a controlled environment, maintained at a consistent temperature (T) ranging from 30 °C to 80 °C, accompanied by a relative humidity of 35% over 6 days. Meanwhile, the second set of specimens was cured for the same duration, yet at a steady temperature of 20 ± 2 °C (SC), complemented by a relative humidity exceeding 95%, within a standard curing box.

On the 7th day, specimens were immersed in water for 24 h with a distance between the water surface and the top of specimens not less than 250 mm. When the specimen is immersed in water, a large number of bubbles and a sizzling sound are produced, indicating that the specimen has a large number of pores that are filled with a large amount of water.

**Figure 5:**
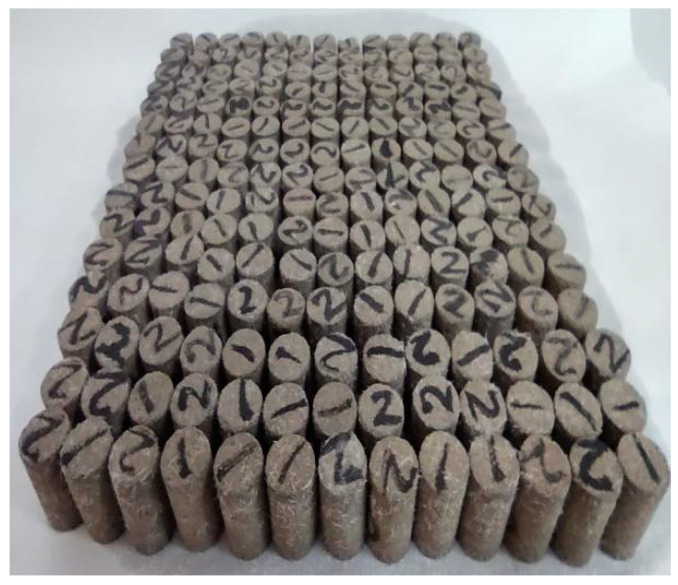
NMR Specimens.

The procedure for preparing the NMR specimen is the same as the procedure for preparing the specimen for the STS test, but the NMR specimen is a cylindrical specimen with a diameter of 20 mm and a height of 30 mm, as shown in Figure 5.

### 2.4. Test Program

**Figure 6:**
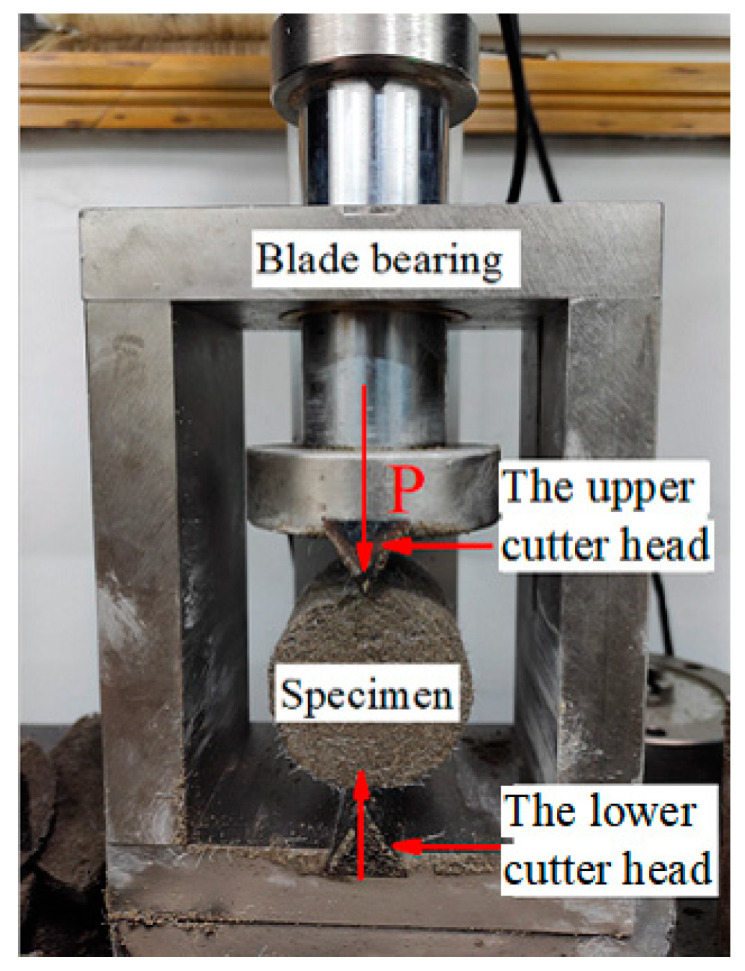
Fixture for splitting tensile strength test.

A comprehensive series of STS assessments was undertaken to delve into the influence of curing temperature on the mechanical characteristics of CSRPF. To facilitate the STS tests, an automated strain-controlled device with a peak load capacity of 5 kN was employed, alongside a dedicated fixture (Figure 6). Following the designated curing period, the specimens were subjected to loading at a controlled rate of 1 mm/min. The loading process continues until the sample is split into two halves. Throughout the entirety of the loading sequence, all examinations were meticulously executed in accordance with the Code for Soil Test of Railway Engineering (TB 10102-2010) [43], a nationally recognized criterion for geotechnical testing within China.

**Figure 7:**
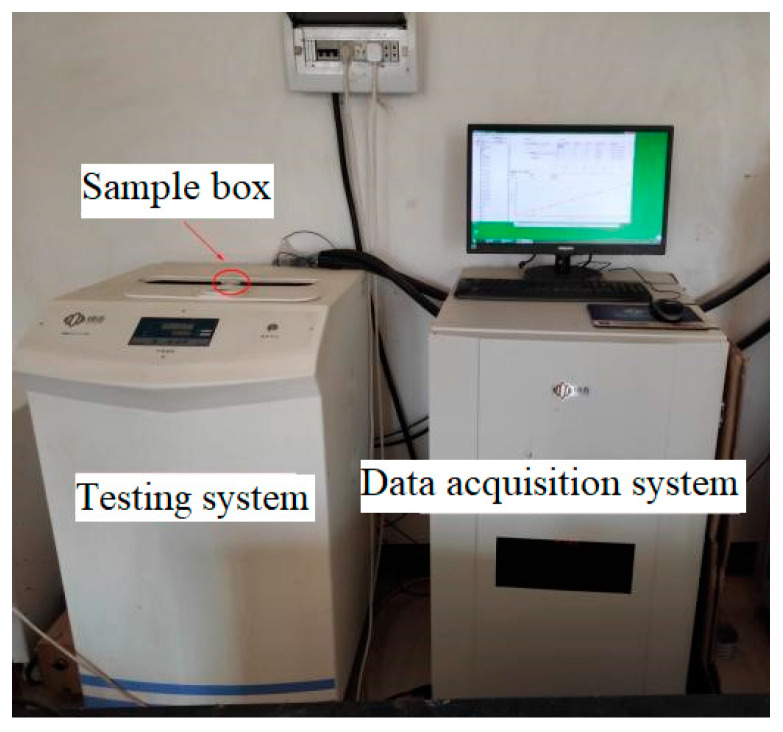
NMRC12-010V Analyzer.

**Figure 8:**
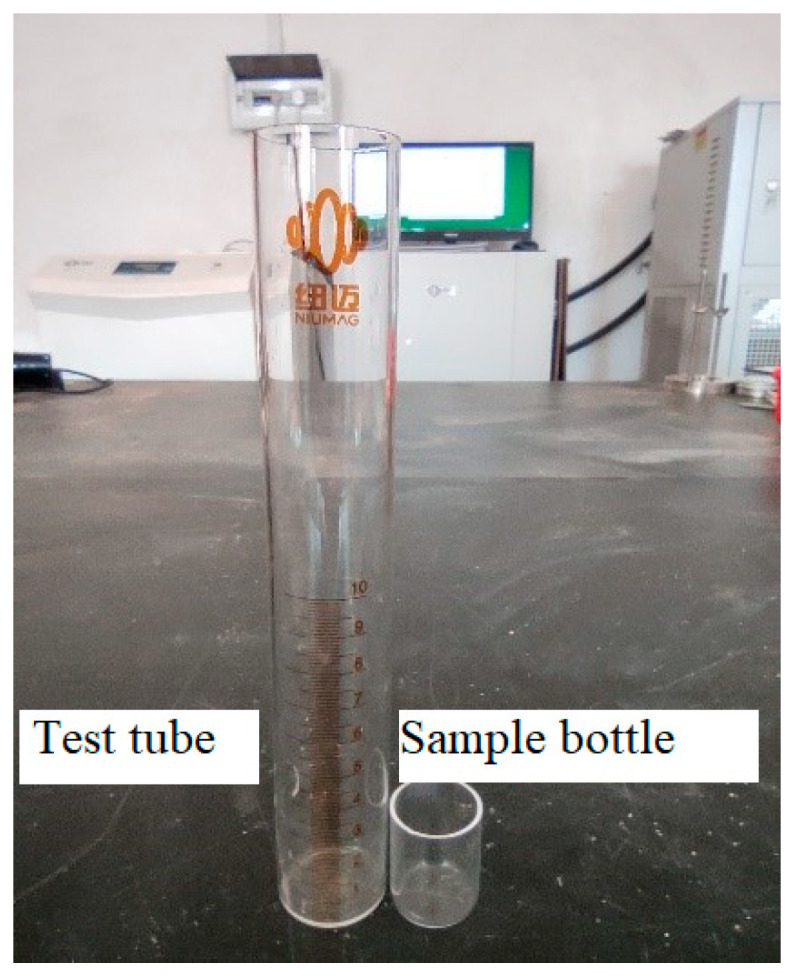
NMR sample bottle and test tube.

Nuclear magnetic resonance (NMR) represents a modern technique for qualitatively assessing the internal pore dimensions of soils. This approach relies on the correlation between the *T*_2_ relaxation time within the hydrogen spectrum and the pore dimensions within a saturated sample [44,45,46]. In comparison to mercury intrusion porosimetry (MIP), NMR offers the advantage of assessing a broader spectrum of pore sizes while maintaining a heightened level of testing precision [47]. In addition, the test method is non-destructive, fast, continuous, repeatable, convenient, and environmentally friendly [48]. The NMRC12-010V Analyzer (Suzhou Niumai Analytical Instrument Co., Ltd., Suzhou, China) was used to measure the pore size distribution of CSRPF at different curing temperatures, as shown in Figure 7. The NMRC12-010V Analyzer consists of the data acquisition system and the test system. Figure 8 shows the NMR test tube and sample bottle. The test procedure was described in detail in the literature [49], and the calculation formula Equation (2) was as follows:

(2) $$\frac{1}{T_{2}}=\rho_{2}{\left(\frac{S}{V}\right)}_{\mathrm{pore}}=\rho_{2}\frac{F_{S}}{r}$$

where *T*_2_ is the transverse relaxation time of fluid in the pore, *ρ*_2_ is the surface relaxation rate, (*S*/*V*) pore is the pore surface area to volume ratio, *F*_S_ is the shape factor of the pore inside the sample, and *r* is pore radius. This study assumed that the internal pores of modified Aeolian sand are cylindrical, so the shape factor *F*_S_ of the internal pores of the sample is 2. According to reference [50], the measured value of transverse surface relaxation strength *ρ*_2_ of cement hydration products is 10 µm/s.

The *T_2_* spectrum curve derived from nuclear magnetic resonance provides insights into the internal pore configuration of the sample. A direct correlation exists between the *T*_2_ relaxation time and the pore radius: longer *T*_2_ relaxation times signify larger pore radii. Additionally, the magnitude of the NMR signal amplitude corresponds to the number of pores associated with a specific pore size, whereby a stronger signal amplitude denotes a higher pore count.

## 3. Results and discussion

### 3.1. Effects of Curing Temperature on T_2_ Spectrum Curves

**Figure 9:**
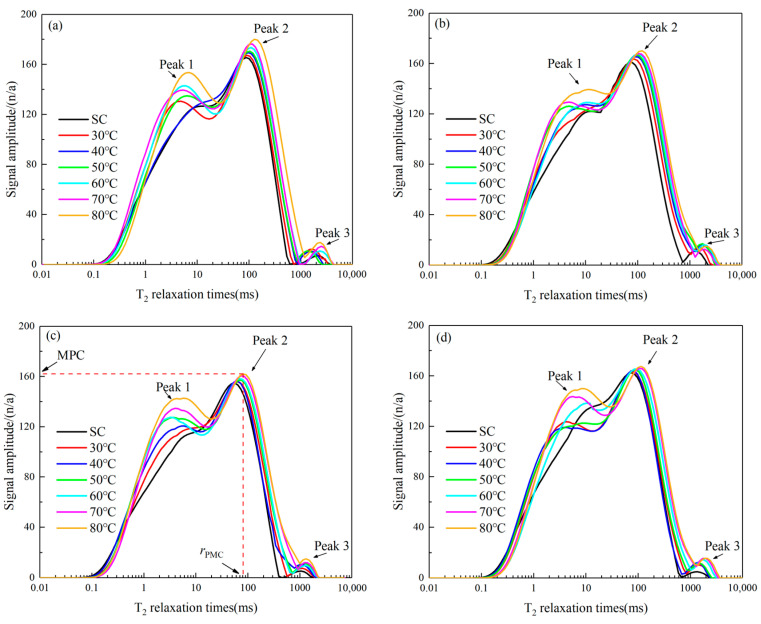
*T*_2_ spectrum curves of CSRPF under different curing temperatures for (**a**) *a_c_* = 5%, *a_f_* = 0‰, (**b**) *a_c_* = 5%, *a_f_* = 6‰, (**c**) *a_c_* = 5%, *a_f_* = 8‰, and (**d**) *a_c_* = 5%, *a_f_* = 10‰.

The nuclear magnetic resonance (NMR) test offers a viable method for establishing the *T*_2_ spectrum curves of CSRPF across diverse curing temperatures. (Figure 9). The *T*_2_ spectrum curves at different curing temperatures are relatively similar, with 2 or 3 relaxation peaks and longer *T*_2_ relaxation times. The *T*_2_ relaxation time has a wide distribution range, with the main distribution range between 0.1 ms and 1000 ms. The curve between Peak 1, Peak 2, and Peak 3 is relatively continuous, and the pore size shows better continuity. However, the peak of Peak 2 in the *T*_2_ spectrum curve and the pore radius corresponding to the peak are quite different under different influencing factors.

The volume fractions of different apertures are obtained by integrating the area under the peak, and the area corresponding to each *T*_2_ relaxation time is approximately the product of the peak and *T*_2_ infinitesimal. Figure 9 shows that the curve of the *T*_2_ spectrum tends to shift to the right with increasing curing temperature. The peak value of peak 2 (maximum pore content (MPC)) gradually increases with the increase in curing temperature [51], and the corresponding radius of maximum pore content (***r*_MPC_**) also gradually increases.

**Figure 10:**
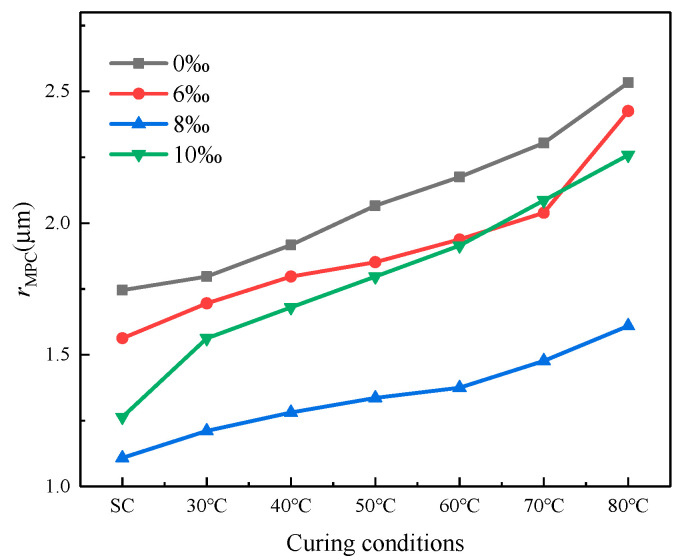
Effect of fiber content and curing temperature on ***r*_MPC_**_._

Figure 10 depicts the evolving trend of ***r*_MPC_** as it relates to variations in both fiber content and curing temperature. With the elevation of curing temperature, a gradual rise in ***r*_MPC_** is observed, indicating that the pore size and pore volume gradually increase. With the increasing curing temperature, a consistent progression is observed in the continuous expansion and bifurcation of microcracks, resulting in the formation of larger pores. Simultaneously, variations in fiber content produce distinct effects: the ***r*_MPC_** value displays an initial increment followed by a subsequent reduction, with the optimal content identified at 8‰. Notably, surpassing this optimum threshold results in incomplete encapsulation of aeolian sand and fiber by the cement matrix, leading to a subsequent increase in ***r*_MPC_**.

### 3.2. Effects of Curing Temperature on Pore Size Distribution

Referring to the pore size classification methods of [52], the pore size distribution of CSRPF is divided into four types of pores based on the pore radius r for analysis, namely micropores (r ≤ 0.01 μm), small pores (0.01 μm < r ≤ 0.1 μm), mesopores (0.1 μm < r ≤ 1 μm), and large pores (r > 1 μm).

**Figure 11:**
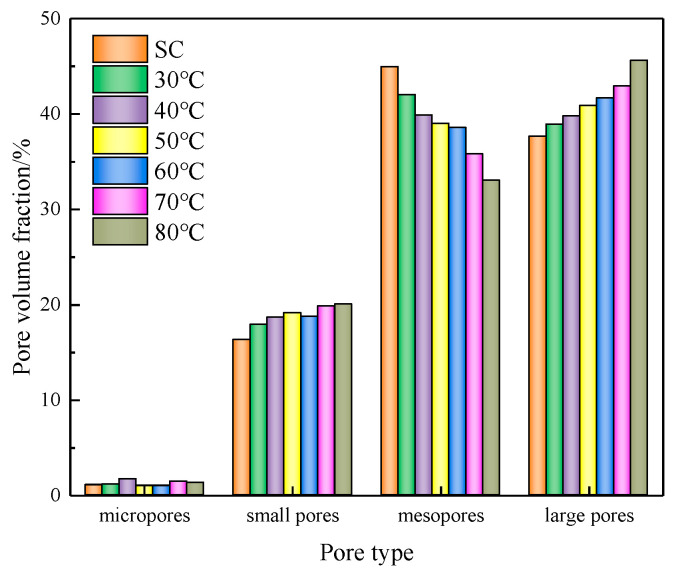
Pore distribution at different curing temperatures.

Figure 11 illustrates the impact of different curing conditions on the pore size distribution of CSRPF, maintaining a fiber content of 8‰. With escalating curing temperatures, there is a concurrent rise in the proportion of small pores and macropores within CSRPF, whereas the proportion of mesopores experiences a decrease. The curing temperature has little effect on micropores.

When the curing temperature is increased from 30 °C to 80 °C, the proportion of small pores and macropores of the CSRPF increases by 2.13% and 5.7%, respectively, while the proportion of mesopores decreases by 9.0%. Under the standard curing conditions, the proportion of mesopores and macropores in CSRPF was 44.9% and 37.7%, respectively, and the proportion of mesopores was the largest. Upon reaching a curing temperature of 80 °C, the composition of macropores within the CSRPF at 45.6% exhibited a notable increase. In contrast, the proportion of mesopores experienced a reduction, reaching 33.0%. As the curing temperature increased, the small pores continued to develop, and a large number of small pores gradually expanded into mesopores. The mesopores then further expanded and connected into large pores and gradually penetrated large, connected cracks. The internal structural damage of the sand increased with the continuous increase in curing temperature.

### 3.3. Effects of Curing Temperature on Stress–Strain Curves

**Figure 12:**
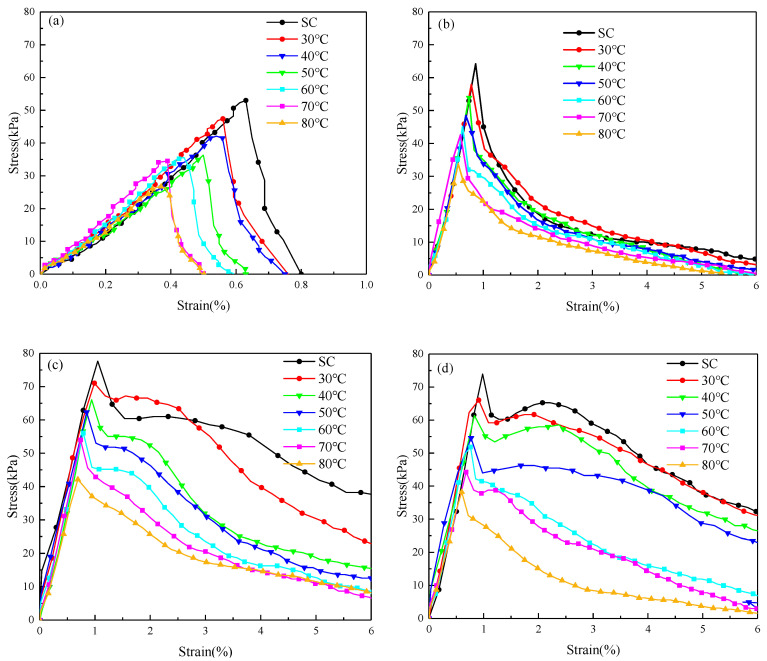
Stress–strain curves of cemented aeolian sand and CSRPF under different curing temperatures for (**a**) *a_c_* = 5%, *a_f_* = 0‰, (**b**) *a_c_* = 5%, *a_f_* = 6‰, (**c**) *a_c_* = 5%, *a_f_* = 8‰, and (**d**) *a_c_* = 5%, *a_f_* = 10‰.

Figure 12 shows selected stress–strain curves of cemented aeolian sand and CSRPF at different curing temperatures. For cemented aeolian sand, a strain of 1% is assumed as the cut-off strain, and for CSRPF, a strain of 6% is assumed as the cut-off strain. The stress–strain curve of CSRPF differs significantly from that of the cemented aeolian sand. Evidently, the curing temperature exerts a noteworthy influence on the stress–strain characteristics of CSRPF. With escalating curing temperatures, the stress–strain curve of CSRPF exhibits a gradual leftward shift, accompanied by a progressive reduction in the peak stress magnitude. In the context of cemented aeolian sand, the rate of stress augmentation during the elastic deformation phase of the stress–strain curve decreases as the curing temperature increases, although the discernible divergence in stress levels at varying curing temperatures remains relatively marginal. The stress of the curve at the higher curing temperature peaks earlier. In the stress–attenuation stage, the maximum strain (the strain at which stress drops to 0) decreases as the curing temperature increases. The elastic deformation phase depicted on the stress–strain curve of CSRPF closely resembles that of the cemented aeolian sand.

### 3.4. Effects of Curing Temperature on STS

**Figure 13:**
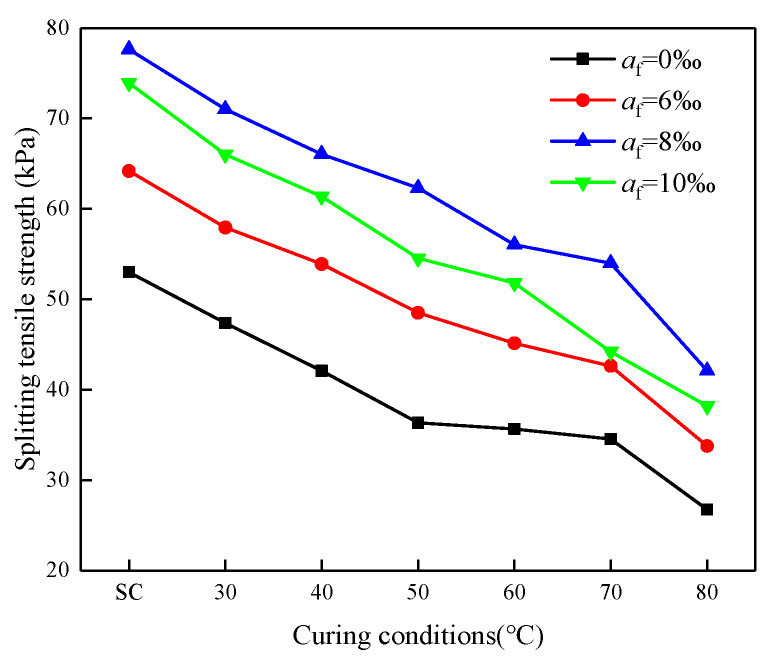
Change in STS at different curing temperatures under different fiber content.

The effects of curing temperature on the development of STS of CSRPF at early ages are shown in Figure 13. With a consistent fiber content of 8‰, elevating the curing temperature from 30 °C to 80 °C leads to a noticeable decline in the Splitting Tensile Strength (STS) of CSRPF, decreasing from 71.0 to 42.1 kPa, which translates into a substantial reduction of 40.7%. This observation underscores a clear inverse relationship between the STS of CSRPF and the curing temperature.

### 3.5. Effects of Fiber Content on STS

**Figure 14:**
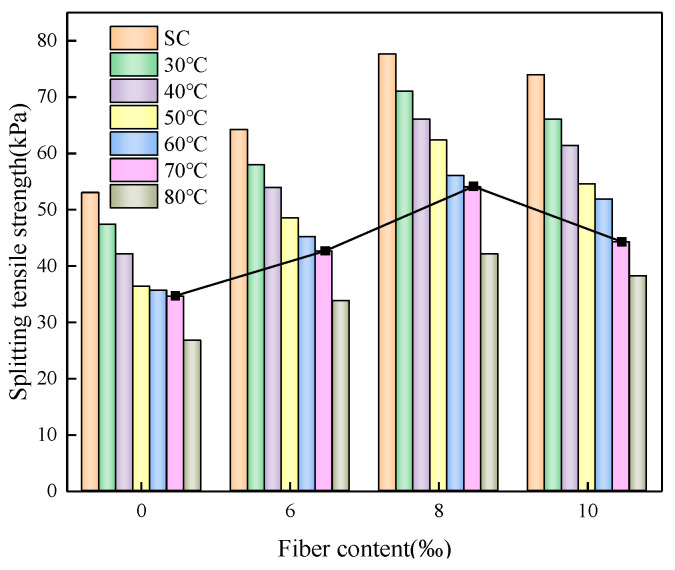
Effects of fiber content on STS.

At a curing temperature of 70 °C, the STS of CSRPF with 0‰, 6‰, 8‰, and 10‰ polypropylene fibers is 34.6 kPa, 42.6 kPa, 54.0 kPa, and 44.2 kPa, respectively. As shown in Figure 14, the addition of polypropylene fiber can significantly improve the STS. This is because the polypropylene fiber bears part of the tensile stress during the deformation of the soil, which can improve the STS and cracking resistance of CSRPF. The high fiber content has a more significant effect on the STS of CSRPF. However, there is an optimum fiber content of 8‰. When the optimum fiber content is exceeded, the STS of CSRPF decreases instead, but the value is still higher than that of cemented aeolian sand. The same trends were observed at all curing temperatures and cement contents. The STS curve is parabolic with the fiber content [53]. The research results of this paper are consistent with this conclusion.

### 3.6. Relationship between **r_MPC_** and STS

**Figure 15:**
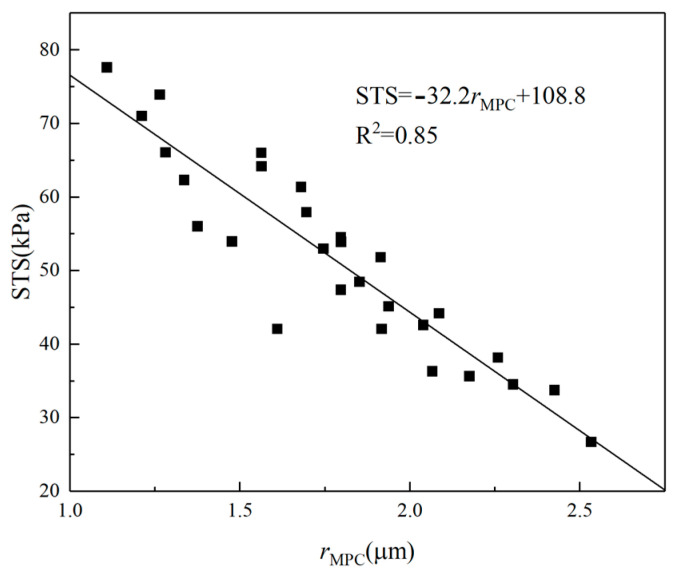
Relationship between *r*_MPC_ and STS.

Figure 15 shows the relationship between ***r*_MPC_** and STS. The STS decreases as ***r*_MPC_** increases. The trends of ***r*_MPC_** and STS with curing temperature and fiber content are consistent.

As the curing temperature rises, it exacerbates the deterioration of the internal structure of CSRPF. Additionally, the elevated temperature induces the evaporation of free water from the sample, leading to a reduction in the available free water necessary for the cement hydration reaction. The cement hydration products are reduced, and the internal pores are increased. The small internal pores gradually expand and connect to form larger pores. Macroscopically, the STS continues to decrease, and the results of the NMR test agree well with the macroscopic mechanical properties. Polypropylene fibers and aeolian sand intricately intertwine, forming a cohesive network that effectively curtails both vertical and lateral deformations within a defined range. This interlocking mechanism not only constrains soil deformation but also amplifies the gripping strength and frictional interactions between the polypropylene fibers and aeolian sand particles [14], and ultimately strengthens the CSRPF. When the optimum fiber content is exceeded, the fibers reduce the cementation of the cement with the aeolian sand. In addition, the unevenly dispersed fibers recombine and prevent the cement from completely enveloping the aeolian sand. This creates a weak interface in the CSRPF, leading to a reduction in STS.

### 3.7. Energy Absorption Capacity

Energy absorption capacity (EAC) stands as a vital attribute within hybrid materials. This property is quantified by the energy encapsulated within the region bounded by the stress–strain curve and the abscissa axis at the reference strain [54]. The cumulative energy absorption is ascertained at a strain level of 6%. The total energy absorption is defined as the energy absorbed before reaching the peak stress plus the residual energy before the cut-off strain.

**Figure 16:**
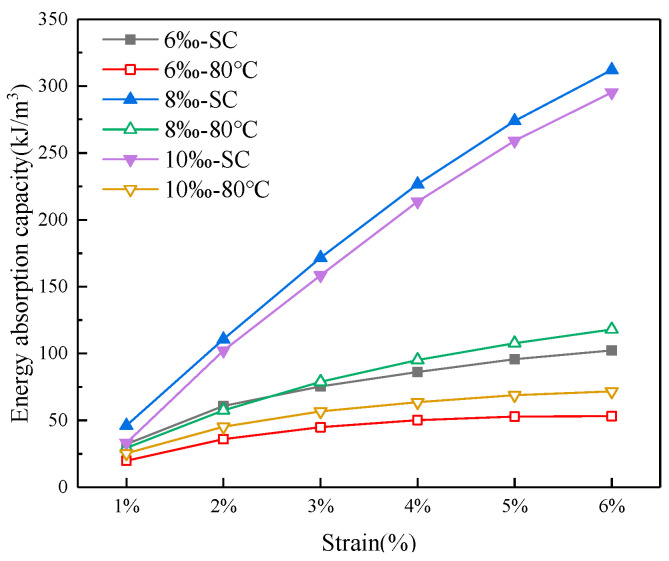
Effect of fiber content on EAC.

As depicted in Figure 16, the energy absorption capacity demonstrates an upward trend with the escalation of strain values beyond 1%. Furthermore, at equivalent strain levels, an augmented fiber content contributes to the enhancement of energy absorption capacity within CSRPF. However, it is noteworthy to mention that elevated temperatures and the utilization of drying curing conditions serve to diminish the overall energy absorption capacity.

**Figure 17:**
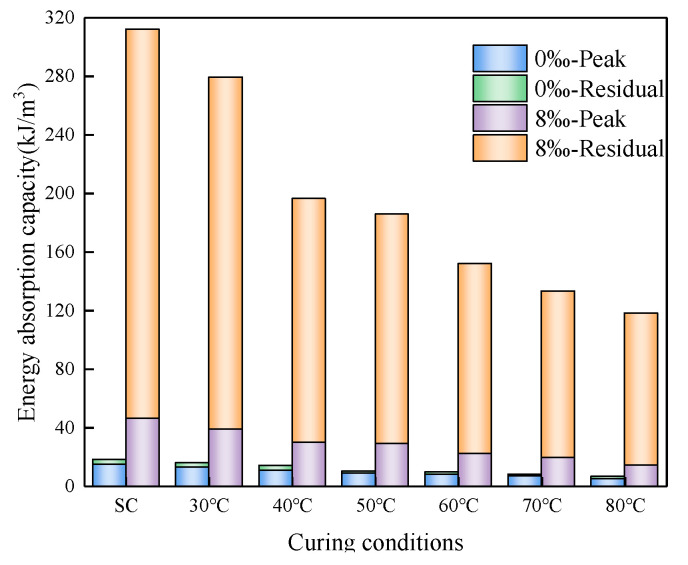
Effect of curing conditions on EAC.

Figure 17 illustrates the energy absorption capacity of CSRPF across various curing conditions, with a consistent fiber content of 8‰. Remarkably, the energy absorption capacity of CSRPF surpasses that of cemented aeolian sand by a significant factor of 5.7, particularly under standard curing conditions. Under the curing condition at high temperatures from 30 °C to 80 °C, the energy absorption capacity of CSRPF was 6.7, 6.2, 8.4, 7.8, 8.7, and 8.4 times that of cemented aeolian sand, respectively. For cemented aeolian sand, the post-peak energy (residual energy) absorption accounts for only 13.7% to 23.9% of the total energy absorption, while energy absorption in the residual stress stage of CSRPF accounts for 76.1% to 84.7% of the total energy absorption. The incorporation of fibers greatly enhanced the energy absorption capacity of cemented aeolian sand, and this enhancement mainly came from the post-peak residual strength.

With the elevation of curing temperature, a noticeable decline in the EAC of CSRPF becomes apparent. Specifically, when the curing temperature is increased from 30 °C to 80 °C, the EAC experiences a reduction of 59.9% for cemented aeolian sand, and 49.3% for CSRPF. This temperature-induced decline can be attributed to increased water evaporation within the specimen, leading to the development of pores. Moreover, the diminished presence of water hampers the advancement of the cement hydration reaction, thereby contributing to the observed decrease in energy absorption capacity.

## 4. Regression Models and Sensitive Analysis

A comprehensive approach involves the utilization of a multivariate nonlinear regression model. The primary objective is to formulate predictive models for the splitting tensile strength (*q*_t_) of CSRPF, wherein *q*_t_ is conceived as a function intricately tied to both curing temperature and fiber content [53]. The form of the regression model, as depicted in Equation (3), is taken into consideration in the current study.

(3) $$q_{t}=k_{0}+k_{1}T+k_{2}a_{f}+k_{3}T^{2}+k_{4}a_{f}{}^{2}+k_{5}Ta_{f}$$

Here, *q*_t_ represents the dependent or output variable, while *T* and *a_f_* represent the independent or input variables, corresponding to curing temperature (°C), and fiber content (‰), respectively. The regression coefficients *k*_0_ to *k*_5_ characterize the coefficients of the regression equation.

The parameter estimates are shown in Table 4. The correlation coefficient of the regression model (*R*^2^) is 0.895, indicating that the reliability of the prediction model is relatively high.

Upon the establishment of regression models, their application was directed toward assessing the influential extent or sensitivity of independent variables, specifically curing temperature and fiber content [55]. The sensitivity was determined using Equations (4) and (5):

(4) $$N_{i}=f_{xi}^{\max}-f_{xi}^{\min}$$

(5) $$S_{i}=\frac{N_{i}}{\sum_{j=1}^{n}N_{j}}\times 100$$

where $f_{xi}^{\max}$ and $f_{xi}^{\min}$ are the maximum and minimum values obtained when the curing temperature and fiber content are input, respectively, when one variable is input, and the other variable equals their mean value; *n* is the number of input variables, which is taken as 2. *S_i_* is the effective degree of the *i*th variable.

According to the sensitivity analysis results, the sensitivity of curing temperature and fiber content on the splitting tensile strengths of CSRPF are 57.7% and 42.9%, respectively. Curing temperature emerges as a pivotal parameter impacting the splitting tensile strength of CSRPF, followed closely by fiber content.

## 5. Conclusions

This study performs a series of splitting tensile strength (STS) tests and nuclear magnetic resonance (NMR) tests to comprehensively investigate the effects of curing temperature on the macro mechanical properties and microscopic pore structure of CSRPF. The main conclusions from the test results can be summarized as follows:

1. The internal pore radius and pore volume of CSRPF samples increase with curing temperature. The ***r*_MPC_** value of CSRPF samples first decreases and then increases with the fiber content increasing, and the optimum fiber content is 8 ‰.
2. The STS of CSRPF samples decreased with increasing curing temperature and increased first and then decreased with increasing fiber content. The results of parameter sensitivity analysis show that curing temperature has a more significant effect on STS of CSRPF.
3. Based on the data fitting of the STS test and NMR test, there is an obvious negative correlation between STS and ***r*_MPC_** of CSRPF under high-temperature curing conditions.

## Figures and Tables

**Table 1:** Physical and mechanical properties of aeolian sand.

Properties	Values	Properties	Values
Density (g/cm^3^)	1.58	Internal friction angle (°)	27.4
Cohesive strength (kPa)	0	Maximum dry density (g/cm^3^)	1.61
Optimum moisture content (%)	12.5	Maximum void ratio	0.92
Specific gravity	2.70	Minimum void ratio	0.59

**Table 2:** Chemical composition of the cement.

Chemical Constituent	Values (%)	Chemical Constituent	Values (%)
CaO	61.8	SO_3_	3.3
SiO_2_	22.1	MgO	2.7
Al_2_O_3_	4.7	LOI	1.6
Fe_2_O_3_	3.8	—	—

**Table 3:** Polypropylene fiber parameters.

Behavior Parameters	Values	Behavior Parameters	Values
Breaking tensile strength (MPa)	486	Dispersibility	Excellent
Length (mm)	6	Elongation at fracture (%)	15~18
Average diameter (µm)	48	Modulus of elasticity (MPa)	>4800
Acid and alkali resistance	Excellent	melting point (°C)	580

**Table 4:** Parameter estimates.

Parameter	Estimated Value	Standard Error	95% Confidence Interval	
			Upper Limit	Lower Limit
*k*_0_	50.951	11.157	27.511	74.391
*k*_1_	−0.139	0.400	−0.978	0.701
*k*_2_	4.132	1.137	1.744	6.520
*k*_3_	−0.002	0.004	−0.009	0.005
*k*_4_	−0.123	0.086	−0.305	0.058
*k*_5_	−0.020	0.014	−0.049	0.009

## Data Availability

Not applicable.
